# Amyotrophic Lateral Sclerosis: Advances and Prospects

**DOI:** 10.3390/jcm12155055

**Published:** 2023-08-01

**Authors:** Matthias Boentert, Andreas Hermann, Julian Großkreutz

**Affiliations:** 1 Department of Neurology with Institute of Translational Neurology, Münster University Hospital, 48149 Münster, Germany; 2 Department of Medicine, UKM Marienhospital Steinfurt, 48565 Steinfurt, Germany; 3 Translational Degeneration Section ‘‘Albrecht Kossel”, Department of Neurology, University of Rostock, 18057 Rostock, Germany; andreas.hermann@med.uni-rostock.de; 4 German Center for Neurodegenerative Diseases Rostock/Greifswald, 18057 Rostock, Germany; 5 Department of Neurology, Precision Medicine, University of Lübeck, 23538 Lübeck, Germany; julian.grosskreutz@neuro.uni-luebeck.de

The *JCM* Topical Collection “Amyotrophic Lateral Sclerosis: Latest Advances and Prospects” started in 2020 and currently includes 11 publications reflecting a broad range of clinical research areas in the ALS field. ALS is a highly complex neurodegenerative disease which is still incurable and inevitably progressive. Advancements in ALS research are continuously being made, and new challenges and opportunities arise with each discovery. Researchers around the world are working diligently to elucidate disease mechanisms, treatment possibilities, and any issues that are relevant for optimized medical and psychosocial care of individuals affected by this devastating disease.

## 1. Patient Care in Times of COVID-19

Modalities and settings of patient care have substantially changed due to the COVID-19 pandemic. In the very first publication in this Topical Collection, Steinbach et al. showed that the D50 model of disease progression, based on the revised ALS Functional Rating Scale, allows the identification of patients with rapid deterioration who should be actively offered advanced and intensified care if regular on-site contact cannot take place [1]. The D50 model stratifies disease severity as a function of time, thus yielding an impression of how aggressively patients are affected and how urgently certain measures of supportive care should be recommended. Detailed comparison of the D50 with other markers of disease progression (e.g., the ΔALSFRS-R) in larger samples will be of interest for future studies.

## 2. Supportive Care

Regarding supportive and palliative care for people with ALS, the involvement and chaperonage of caregivers is crucial. Linse et al. present an observational study focusing on caregivers’ needs and perceptions [2]. Although specifically representing the situation in Germany, this study highlights that the provision of customized technical aids and home nursing are major issues directly impacting the quality of life of affected families. Two publications in the *JCM* Topical Collection, both from the same group, focus on pain in people with ALS. Based on various patient-reported outcome measures, the authors were able to show that pain (of whatever etiology) is highly prevalent but underrecognized in ALS patients [3]. Pain severely impacts health-related quality of life and well-being. The study results underpin that pain and depression are closely related to each other in ALS patients, requiring therapeutic awareness throughout the course of the disease [4].

Cognitive and behavioral impairment is apparent in approximately 50% of ALS cases, and is diagnosed and monitored using standardized cognitive function tests. These are mostly paper-and-pencil-based, and thus may be impeded by motor impairment. In an interesting study, Schmitz-Peiffer et al. validated an eye-tracking computer-based cognitive test in subjects with ALS and Parkinson’s disease [5]. Since an accurate assessment of cognitive problems is important for both patient counselling and adequate treatment decisions, the proposed test may fill a practical gap in clinical practice.

## 3. Respiratory Muscle Involvement

Respiratory muscle involvement eventually occurs in most patients with ALS and is a major determinant of overall prognosis [6]. In ALS and spinal muscular atrophy (SMA), non-invasive mechanical ventilation has proven to positively influence both quality of life and survival rates [7,8]. Thus, the early diagnosis of inspiratory muscle weakness and hypoventilation are mainstays of optimal patient care. The work by Hermann et al. focuses on ultrasound as a bedside tool to assess diaphragm function in patients with ALS and SMA, underlining that ultrasound findings may specifically differ depending on the underlying neuromuscular condition [9]. Considering the prognostic impact of diaphragm weakness, inspiratory muscle strength training (IMST) in ALS has been evaluated by a limited number of studies, including the original article in this collection by Vicente-Campos et al., which reports results from a prospective case–control study [10]. Participants performed IMST against a resistive load over an eight-week period, resulting in a significant improvement in maximum inspiratory pressure. As a recent randomized trial involving people with early-stage ALS showed an improvement in expiratory but not inspiratory muscle strength [11], further research is necessary to identify optimal training regimens for patients with variable disease severity and distinct ALS phenotypes. 

## 4. Phenotypic Variability of ALS

The retrospective cohort study by Requardt et al. adds to the growing body of evidence that in ALS, overall prognosis and survival are immanently codetermined by manifestation age, progression rate, and disease phenotype [12]. This study is in line with previous reports on the role of specific phenotypes (i.e., bulbar ALS or ALS-FTD) [13,14,15] and underscores the fact that phenotypic variability must be recognized in future clinical trials if precision therapies shall be made available for distinct patient subgroups [16].

## 5. Treatment Strategies

Developing effective disease-modifying therapies that can halt or reverse motor neuron loss are a major focus of ALS research. ALS is likely to represent a complex interplay of genetic, environmental, and immune factors. While the latter are still not fully understood, targeting immune-mediated mechanisms has emerged as a potential therapeutic strategy. In this context, Sobus et al. present results from an open-label interventional trial that intrathecally administered autologous bone-marrow-derived lineage-negative early hematopoietic cells [17]. These cells were of interest since the group has shown in previous studies that this cell population produces high amounts of neurotrophic factors. Interestingly, this approach alleviated complement activation by reducing plasma C3b concentrations.

## 6. ALS Genetics

Two publications in the Topical Collection focus on the genetic background of ALS or ALS pathogenesis, respectively. Yilihamu et al. investigated whether pathogenic mutations in the sorbitol dehydrogenase (SORD) gene are prevalent in a large cohort of Chinese patients with ALS [18]. This approach was stimulated by the fact that a homozygous mutation in the SORD gene was recently found in a French patient with juvenile-onset ALS [19]. As the authors did not find pathogenic variants in 601 patients with sporadic ALS, exploratory analysis of the SORD gene in other ethnicities and in patients with unexplained familial ALS will help to separate uncommon ALS phenotypes from SORD-related motor neuropathies [20]. 

Despite the broad range of hypotheses about the molecular causes of familial and sporadic ALS, the exact disease mechanisms and their interplay remain unclear. Since incidence rates differ between different ethnicities, the multigenetic etiology of sporadic ALS may vary among distinct populations. D’Antona et al. systematically investigated single-nucleotide polymorphisms (SNPs) in ALS-related genes while comparing different European populations [21]. Using a computational approach, the authors were able to identify various SNPs and SNP-related genes that may account for regional differences in ALS frequency in Europe, and subsequent exploration of these findings may enhance our understanding of disease mechanisms and potential treatment strategies.

## 7. Conclusions

Various aspects from the ALS field are represented in the *JCM* Topical Collection “Amyotrophic Lateral Sclerosis: Latest Advances and Prospects”, including articles that open new prospects for both clinical and molecular research in the future.
